# Joint Inversion of Radionuclide Production Rate Data and Thermoremanent Magnetic Records over the Holocene

**DOI:** 10.1007/s11207-025-02559-0

**Published:** 2025-11-06

**Authors:** Maximilian Arthus Schanner, Andreas Nilsson, Raimund Muscheler

**Affiliations:** 1 GFZ Helmholtz Centre for Geosciences, Section 2.3 Geomagnetism, Potsdam, Germany; 2 Department of Geology, Lund University, Lund, Sweden

**Keywords:** Magnetic fields, models, Cosmic rays, galactic, Solar cycle, models

## Abstract

Understanding the Sun’s role in past climate change requires knowledge of solar variability over millennia. While direct sunspot records span only the last 400 years, longer-term changes are inferred from cosmogenic radionuclides like ^14^C and ^10^Be in tree rings and ice cores. Their production reflects variations in galactic cosmic ray flux, modulated by Earth’s and Sun’s magnetic fields - the latter is tied to solar activity. We present a Bayesian model that jointly reconstructs solar modulation and the global geomagnetic field over the Holocene. Extending previous work, our model directly incorporates ^14^C and ^10^Be production rate data and thermoremanent magnetic records. A flexible prior allows for bimodality and explicit long-term trends in solar activity. The reconstruction shows a clear separation of grand solar minima and a normal mode. Additionally, we explore the recovery of an 11-year cycle in solar modulation.

## Introduction

The Sun is the main source of energy in the solar system (e.g. Bard and Frank 2006). Solar variability has been suggested to generate climatic changes in the past (e.g. Bond et al. 2001; Owens et al. 2017), recent times, and possibly the future (Feulner and Rahmstorf 2010). Assessment of this connection on longer timescales requires reconstructions of solar variability and the climate. For the latter, different proxies like $\delta$ ^18^O, pollen, and many more exist, while the former can be observed through the abundance of cosmogenic radionuclides like ^14^C and ^10^Be. Records of these radionuclides can be obtained from tree rings and ice cores.

The production rates of these radionuclides depend on the solar and the Earth’s magnetic fields (Potgieter 2013; Herbst, Kopp, and Heber 2013). Therefore, their production rates contain an entangled signal of the two processes. Other signals, like variations of the carbon cycle for ^14^C and transport and deposition effects for ^10^Be in ice cores, are contained in the records as well, but are of smaller magnitude during the investigated time period and therefore not considered in this study (see for example Muscheler et al. 2004). One challenge in inferring the evolution of solar variability is separating the various signals contained in the production rate records. This has led to disagreeing reconstructions of solar activity in the past (Solanki et al. 2004; Muscheler et al. 2005; Vonmoos, Beer, and Muscheler 2006; Steinhilber et al. 2012). Due to the limited data over the Holocene and lack of detailed knowledge regarding the effects of the carbon cycle and radionuclide transport changes, a Bayesian prior informed by outside knowledge can help significantly with this challenge.

Nilsson et al. (2024) developed a probabilistic model for the solar variability over the Holocene. The model also includes the geomagnetic field, but the magnetic data are not modeled directly. Instead, different global field models are included as input data (Nilsson et al. 2022; Schanner, Korte, and Holschneider 2022). Nevertheless, their model is able to fit several Holocene cosmogenic isotope records by considering hemispherical asymmetries in their production rates. They solve a long-standing discrepancy between Greenland ^10^Be and tree ring ^14^C data and find no compelling evidence for long-term variations in the solar variability, apart from clustering of grand solar minima. Additionally, their results hint at a hemispherical bias in the included global geomagnetic field models.

To investigate the joint influence of solar variability and the global geomagnetic field on radionuclide production rates further, and to evaluate what information about the two processes can be inferred from the production rate records, we provide an extended version of the probabilistic model of Nilsson et al. (2024). The major difference is that we model the global geomagnetic field alongside the solar variability, i.e. data from thermoremanent records (GEOMAGIA database; Brown et al. 2015) are included directly in the modeling procedure. In addition, we use a more flexible prior for the solar component, to reflect the possibility of a bimodal distribution in solar activity, which may arise due to grand solar minima (Usoskin et al. 2014; Wu et al. 2018; Nilsson et al. 2024). We investigate the presence of a long-term trend in the solar modulation and explore the recovery of an 11-year cycle over the last 3000 years by explicitly including respective terms in the prior. Our findings are presented in the Section 4. A detailed description of the prior and the modeling procedure is given in the Section 2. The data used is described in Section 3.

## Modeling

Our statistical model is embedded in a Bayesian setting and can be split into three components. The paleomagnetic component contains a Gaussian process prior for the global Holocene geomagnetic field. The solar component consists of a flexible prior for the solar variability, that allows bimodality, long-term evolution, and an 11-year cycle. Finally, the data model consists of two likelihood terms. One term, for the thermoremanent magnetic records, is only connected to the geomagnetic field, while the other term, describing the radionuclide production data, is connected to the solar variability and the dipole and axial quadrupole of the geomagnetic field. All components are implemented in the probabilistic programming language PyMC (Python Monte-Carlo, Abril-Pla et al. 2023). The posterior is inferred via a Hamiltonian Monte-Carlo sampling. We present each component and the sampling metrics in detail in the following sections.

### Paleomagnetic Component

The prior for the global geomagnetic field is the same as the one proposed by Nilsson et al. (2022). The field is modeled in terms of Gauss coefficients up to degree 5. A priori, the Gauss coefficients are uncorrelated with degree-dependent variances given in Table 1. The temporal evolution is described by a multivariate Gaussian process with a Matérn- kernel and degree-dependent correlation times, also given in Table 1. The axial dipole is modeled separately, with a constant mean of − 32.5 $\mu$T and a two-parameter covariance function, similar to the one proposed by Hellio and Gillet (2018) (see also Bouligand et al. 2016):

1 $$ k_{g_{1}^{0}}(\Delta t) = \frac{1}{2\xi _{1}^{0}} \Big( \big(\chi _{1}^{0} + \xi _{1}^{0}\big)\exp \big[- \big(\chi _{1}^{0}-\xi _{1}^{0}\big)\Delta t\big] - \big(\chi _{1}^{0} - \xi _{1}^{0}\big)\exp \big[-\big(\chi _{1}^{0}+\xi _{1}^{0}\big)\Delta t\big]\Big)~, $$

where $\Delta t = \big|t-t'\big|$ is the time lag between two epochs $t$ and $t'$. $\xi _{1}^{0}$, $\chi _{1}^{0}$ and $\omega _{1}^{0}$ are correlation times, with $\xi _{1}^{0}$ given by the other two via $(\xi _{1}^{0})^{2} = (\chi _{1}^{0})^{2} - (\omega _{1}^{0})^{2}$. The values of the two independent ones are given in Table 1. The kernel function gives rise to a stochastic process with two different regimes in the temporal power spectrum. At low frequencies $f$ it is flat, at intermediate frequencies it behaves as $f^{-2}$ and at high frequencies it behaves as $f^{-4}$. See Bouligand et al. (2016) for additional details.

**Table 1:** Parameters of the Gaussian process prior for the global geomagnetic field, similar to those suggested by Nilsson et al. (2022). The values are chosen to facilitate comparison to Nilsson et al. (2024).

Coefficients	Std. deviation [*μ*T]		Correlation time [yrs.]	
$g_{1}^{0}$	$\sigma _{1}^{0}$	10.00	$(\omega _{1}^{0})^{-1}$	741
			$(\chi _{1}^{0})^{-1}$	138
$g_{1}^{\diamond}$	$\sigma _{1}^{\diamond}$	3.50	$\tau _{1}^{\diamond}$	200
$g_{2}^{\diamond}$	$\sigma _{2}^{\diamond}$	1.77	$\tau _{2}^{\diamond}$	133
$g_{3}^{\diamond}$	$\sigma _{3}^{\diamond}$	1.01	$\tau _{3}^{\diamond}$	174
$g_{4}^{\diamond}$	$\sigma _{4}^{\diamond}$	0.46	$\tau _{4}^{\diamond}$	138
$g_{5}^{\diamond}$	$\sigma _{5}^{\diamond}$	0.18	$\tau _{5}^{\diamond}$	95

To accelerate the sampling procedure, we approximate the Gaussian process by a piecewise linear function over fixed intervals of 50 years width. The 50 years of temporal resolution are appropriate for the resolution that is expected from Holocene magnetic data (e.g. Schanner, Korte, and Holschneider 2022). The prior at the knot points is given by the covariance of the Gaussian process, evaluated at those points. This reduces the infinite dimensional Gaussian process space to a multivariate normal distribution. A knot spacing of 50 years is still sufficient to capture the variability that is expected to be resolvable from Holocene geomagnetic data.

Similar to Nilsson et al. (2022), information from the historical and satellite era is incorporated into our model. At the three most recent knots (1900 CE, 1950 CE and 2000 CE), the Kalmag model (Baerenzung et al. 2022) is treated as noise free observations and one inversion step on the multivariate normal distribution is performed. This way, the prior model is “glued” to the present day field.

### Solar Component

We impose the typical, simplified force-field approximation and model the solar variability by a single modulation parameter $\phi $ (Gleeson and Axford 1968). For the production rate calculations (Equations 13 – 16), we use the local interstellar spectrum (LIS) of Burger, Potgieter, and Heber (2000), parameterized by Usoskin et al. (2005). A detailed description of this parameterization can be found in the Appendix of Nilsson et al. (2024). Multiple reconstructions of the solar modulation over the Holocene found hints of bimodality in the histogram (Steinhilber et al. 2012; Wu et al. 2018; Nilsson et al. 2024). This is likely due to grand solar minima, during which the solar dynamo likely operates in a different mode (see for example Kitchatinov and Olemskoy (2010) or Karak (2023) for a review). The different dynamo state results in significantly lower solar shielding due to a weaker magnetic field and very few observable sunspots, such as during the Maunder minimum. To reflect the possible bimodality in the prior, we model the solar modulation $\phi $ as follows.

The starting point is a piecewise linear function, with a multivariate Gaussian prior for the function values at the knot points. This is similar to the Gaussian process used to model the geomagnetic field. We choose a knot spacing of 22 years and a Matérn- kernel with correlation time $\tau _{\text{GP}} = 25.6$ years. The knot spacing is chosen to agree with the epochs of the 22-year averaged data (see Section 3 for additional information). The correlation time is chosen to agree with a solar modulation reconstruction from neutron monitor data (Nguyen et al. 2022). The slightly shorter value, 25.6 compared to $\tau _{GP}=27.2$ years used by Nilsson et al. (2024), is due to a different normalization of the neutron monitor data used to calculate it (see Nilsson et al. 2024), but we find that this difference does not influence the main results noticably.

A scale of $\sigma _{\text{GP}} = 191$ MV and a constant mean of $\mu _{\text{GP}} = 550$ MV are assumed. These are necessary to “glue” the linearized Gaussian process to modern day $\phi $ for the five most recent knots, just like the geomagnetic field is connected to the Kalmag model. The model is later standardized (mean is subtracted and the model is divided by the scale), so that the parameters $\mu _{\text{GP}}$ and $\sigma _{\text{GP}}$ only influence the model during the “glueing”. We test this by changing the values and find indeed no influence on the final model that could be distinguished from fluctuations due to the sampling nature of the modeling approach. At the five most recent knots (from 1934 to 2022), modern day reconstructions of the solar modulation parameter $\phi $ (see Nguyen et al. 2022) are obtained from neutron monitor data (Usoskin, Bazilevskaya, and Kovaltsov 2011) and used for the “glueing”.

At each knot point $t_{\text{knot}}$, $\phi $ is normally distributed

2 $$ \phi _{\text{GP}}(t_{\text{knot}}) \sim \mathcal{N} (\mu (t_{\text{knot}}), \sigma (t_{\text{knot}}))~, $$

where $\mu (t_{\text{knot}})$ and $\sigma (t_{\text{knot}})$ stem from the posterior of a single inversion with the modern day reconstructions of $\phi $. While $\phi (t)$ is pointwise normally distributed, for any number of time points, the $\phi (t_{1}),\dots \phi (t_{n})$ are correlated through the single inversion posterior covariance function. Next, the correlated function values at the knot points are transformed from a multivariate normal distribution to a correlated, pointwise bimodal distribution via inverse cumulative density sampling. Therefore, the knot point distributions are standardized by subtracting the a priori mean $\mu _{\text{GP}}$ and dividing by the prior scale $\sigma _{\text{GP}}$.

3 $$\begin{aligned} \phi _{\text{standard}}(t_{\text{knot}}) =& \frac{\phi _{\text{GP}}(t_{\text{knot}}) - \mu _{\text{GP}}}{\sigma _{\text{GP}}} \end{aligned}$$

4 $$\begin{aligned} \sim & \mathcal{N} \bigg(\mu (t_{\text{knot}}) - \mu _{\text{GP}}\approx 0, \frac{\sigma (t_{\text{knot}})}{\sigma _{\text{GP}}}\approx 1\bigg) \end{aligned}$$

The standardized random variables are then transformed to approximately uniform distributions, by applying the cumulative density function (cdf) of the standard normal distribution:

5 $$ \phi _{\text{uniform}}(t_{\text{knot}}) = \frac{1}{2} \bigg[1 + \text{erf}\bigg(\frac{\phi _{\text{standard}}(t_{\text{knot}})}{\sqrt{2}}\bigg)\bigg] $$

Here $\text{erf}$ is the error function. The inverse distribution $F^{-1}$ of a flexible (i.e. possibly bimodal) random variable can be constructed from the weighted sum of two beta cdfs:

6 $$ \begin{aligned} F^{-1}(x) =& \phi _{\text{lower}} + \phi _{\text{range}} \\ &\cdot \bigg((1 - \kappa ) I_{x}(\alpha _{1}, \beta _{1}) \\ &+ \kappa I_{x}(\alpha _{2}, \beta _{2}) \bigg)~. \end{aligned} $$

**Figure 1:**
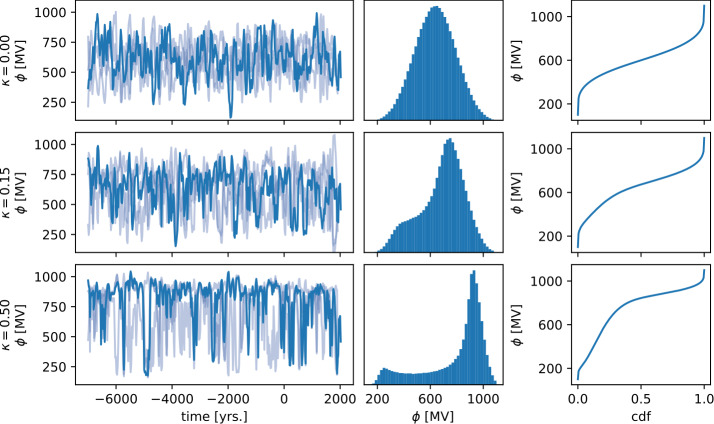
The prior for the solar modulation with different choices of $\kappa $ (top row: $\kappa =0$, center row: $\kappa =0.15$, bottom row: $\kappa =0.5$). The left column depicts four samples from the prior. One sample per row is highlighted and drawn opaque. The center column shows the histogram of all samples at all knot points. With increasing $\kappa $, the increasing bimodality is clearly visible. The right column depicts the inverse cdf, used for generating the samples. See the text for additional details. For these plots, $\phi _{\text{lower}}=100$ MV and $\phi _{\text{range}}=1000$ MV are considered fixed.

$\phi _{\text{lower}}$ is a lower bound for the solar modulation, $\phi _{\text{range}}$ scales the inverse cdf and $\kappa $ controls the amount of bimodality in the prior. These parameters are random variables in our model (hyperparameters), i.e. they will be determined during the inference (see Table 2). $I_{x}(\alpha , \beta )$ is the beta cdf (the regularized, incomplete beta function). Examples of $F^{-1}$ for different values of $\kappa $ are provided in the right column of Figure 1. The prior for the solar modulation at the knot points is given by

7 $$ \phi (t_{\text{knot}}) = F^{-1}(\phi _{\text{uniform}} (t_{\text{knot}}))~. $$

**Table 2:** Parameters and hyperparameters of the solar modulation prior. $\mathcal{HN}$ stands for the half normal distribution and $\mathcal{TN}$ for the truncated normal distribution. The Gamma distribution is parameterized by shape $\alpha $ and rate $\beta $.

Parameter	$\alpha _{1}$	$\beta _{1}$	$\mu _{\text{GP}}$	$\sigma _{\text{GP}}$
Value	0.25	0.25	550 MV	191 MV
Parameter	$\alpha _{2}$	$\beta _{2}$	$\tau _{\text{GP}}$	
Value	2.5	10	25.6 yrs.	
Parameter	$\sigma _{\text{11-year}}$	$p_{\text{11-year}}$	$\tau _{\text{11-year}}$	
Value	250 MV	10.4 yrs.	20 yrs.	

Hyperparameter	Distribution
$\phi _{\text{lower}}$	$\mathcal{HN}(0, 200~\text{MV})$
$\phi _{\text{range}}$	$\mathcal{TN}(600~\text{MV}, 200~\text{MV}; 0, \infty )$
*κ*	Beta(*a* = 1,*b* = 7)
$\sigma _{\text{long-term}}$	
$\tau _{\text{long-term}}$	

Samples of the a priori process for different values of $\kappa $ are shown in Figure 1. In order to explicitly include long-term variability, we add a zero mean Gaussian process with varying correlation times $\tau _{\text{long-term}}$ to the solar modulation prior. The scale $\sigma _{\text{long-term}}$ of this Gaussian process is included as a hyperparameter (see Table 2). To model the 11-year cycle, we also add a zero mean Gaussian process to the prior. This process has a quasi-periodic cosine-squared kernel of the form

8 $$ k_{\text{11-year}}(\Delta t) = \sigma _{\text{11-year}}^{2} \cos\bigg(\frac{\pi \cdot |\Delta t|}{p_{\text{11-year}}}\bigg)^{2} \exp\Bigg(-\frac{1}{2} \bigg(\frac{\Delta t}{\tau _{\text{11-year}}}\bigg)^{2}\Bigg)~. $$

$\sigma _{\text{11-year}}$ is the a priori scale of the process and $p_{\text{11-year}}$ and $\tau _{\text{11-year}}$ are its period and correlation time, respectively. We choose a quasi-periodic kernel instead of a strictly periodic kernel to allow for variations in phase, duration, and amplitude over time. This 11-year cycle component is connected only to ^14^C data from the last 3000 years. We list the prior parameters of the solar component in Table 2. To ensure, that the solar modulation $\phi $ is positive, we add a soft sigmoid constraint to the prior:

9 $$ \log p_{\text{constraint}}(\phi ) = \log(\text{sig}(0.01 \cdot (\phi - 150~\text{MV})))~, $$

where $\text{sig}(x) = 1 / (1 + \exp(-x))$ is the logistic sigmoid function.

### Data Model

The likelihood in our model consists of two terms. Observations of the geomagnetic field are provided as directions (declination $D$ and/or inclination $I$) or intensity $F$. All of these relate non-linearly to the magnetic field vector (see e.g. Hellio et al. 2014). The magnetic field vector itself relates to the Gauss coefficients via spherical harmonics basis functions $S(x)$. For every point $x$ in space and $t$ in time, an observation of the geomagnetic field is given by

10 $$ o_{\text{mag}}(x, t) = H[S(x)\cdot \boldsymbol{m}(t)] + \epsilon _{ \text{mag}}~, $$

where $H\in \{D, I, F\}$, $\boldsymbol{m}(t)$ is a vector of Gauss coefficients $g_{\ell}^{m}(t)$, interpolated from the prior as described above, and $\epsilon _{\text{mag}}$ is a measurement error term. We model

11 $$ \epsilon _{\text{mag}}\sim \mathcal{T}_{4}(0, \sigma _{H})~, $$

as a Student’s $\mathcal{T}$-distribution with 4 degrees of freedom and scale parameter given by the reported measurement error $\sigma _{H}$. Additionally, the observation times $t$ are not known precisely, but modeled as normally distributed random variables

12 $$ t\sim \mathcal{N}(\mu _{t}, \sigma _{t})~, $$

where $\mu _{t}$ are the published ages and $\sigma _{t}$ the reported dating errors.

For the radionuclide production rates, we use the same data model as Nilsson et al. (2024). The production rates depend on the geomagnetic field and the solar modulation via production rate models. For ^14^C, we use a quadratic approximation to the production surface of Kovaltsov, Mishev, and Usoskin (2012):

13 $$\begin{aligned} Q_{\text{GL}}^{\text{14C}}(\text{DM}, \phi ) =& \bigg(9.06 \cdot 10^{-2} + 3.12\cdot 10^{-2} \cdot \text{DM} + 2.62\cdot 10^{-4} \cdot \phi -1.02\cdot 10^{-4}\cdot \text{DM}^{2} \\ & + 1.91\cdot 10^{-5}\cdot \phi \cdot \text{DM}+1.07\cdot 10^{-8} \cdot \phi ^{2}\bigg)^{-1} \end{aligned}$$

$\text{DM} = 0.63712^{3} \cdot \sqrt{(g_{1}^{0})^{2}+(g_{1}^{1})^{2}+(g_{1}^{-1})^{2}}$ is the geomagnetic dipole moment, calculated from the first three entries of the vector $\boldsymbol {m}$ (see Equation 10). The time dependence of $\text{DM}$ and $\phi $ is omitted for brevity. For ^10^Be, the global production rate model of Kovaltsov and Usoskin (2010) is approximated using a quadratic surface:

14 $$ \begin{aligned} Q_{\text{GL}}^{\text{10Be}}(\text{DM}, \phi ) =& \bigg(5.58 + 1.90\cdot \text{DM}+1.38\cdot 10^{-2}\cdot \phi - 1.30\cdot 10^{-2} \cdot \text{DM}^{2} \\ &+1.49\cdot 10^{-3} \cdot \phi \cdot \text{DM} - 2.85\cdot 10^{-7} \cdot \phi ^{2}\bigg)^{-1} \end{aligned} $$

We model the arctic and antarctic records of ^10^Be production rates separately, each as the average of the respective hemisphere (northern, NH and southern, SH) (see Nilsson et al. 2024; Zheng et al. 2024)

15 $$\begin{aligned} Q_{\text{NH}}^{\text{10Be}}(\text{DM}, g_{2}^{0}, \phi ) =& \bigg(1 + \frac{\text{hpa}(\phi , g_{2}^{0})}{2}\bigg) Q_{\text{GL}}^{ \text{10Be}}(\text{DM}, \phi ) \end{aligned}$$

16 $$\begin{aligned} Q_{\text{SH}}^{\text{10Be}}(\text{DM}, g_{2}^{0}, \phi ) =& \bigg(1 - \frac{\text{hpa}(\phi , g_{2}^{0})}{2}\bigg) Q_{\text{GL}}^{ \text{10Be}}(\text{DM}, \phi ) \end{aligned}$$

The asymmetry in production is related to the axial quadrupole of the geomagnetic field $g_{2}^{0}$ and the solar variability. Similar to Nilsson et al. (2024), we use a third order polynomial in $\phi $, to model the proportionality of hemispherical production rate asymmetry ($\text{hpa}$) and geomagnetic field:

17 $$\begin{aligned} &\text{hpa}(\phi , g_{2}^{0}) \\ &\quad = g_{2}^{0}\cdot \big(-1.06\cdot 10^{-15}\cdot \phi ^{3} + 6.11\cdot 10^{-12}\cdot \phi ^{2} - 1.99\cdot 10^{-8}\cdot \phi + 6.07\cdot 10^{-5}\big) \end{aligned}$$

The radionuclide production rate observations are then modeled as

18 $$ o_{\text{rad}}(x, t) = s_{\diamond}\cdot Q_{\diamond}(x, t) + \epsilon _{\text{rad}}~, $$

where $Q_{\diamond}\in \big\{Q_{\text{GL}}^{\text{14C}}, Q_{\text{NH}}^{\text{10Be}}, Q_{\text{SH}}^{\text{10Be}}\big\}$ and $\epsilon _{\text{rad}}\sim \mathcal{N}(0, \sigma _{\text{rad}})$. $\sigma _{\text{rad}}$ is assigned a 5% error for ^14^C and a 10% error for ^10^Be, based on typical values. For each class of production rates (global ^14^C, northern and southern hemisphere ^10^Be), $s_{\diamond}\sim \mathcal{N}(1,0.05)$ is an additional factor, addressing uncertainty in the calibration of the data.

### Hamiltonian Monte-Carlo with PyMC

The non-linear data model makes the full posterior distribution inaccessible in closed form. The geomagnetic field part can be linearized (e.g. Schanner, Korte, and Holschneider 2022), but for the production rates, this is not straight forward. Instead of closed form inference, we utilize Hamiltonian Monte-Carlo inference, to generate an ensemble of posterior samples (e.g. Betancourt 2018). Therefore, we implement the model in the probabilistic programming language PyMC (Abril-Pla et al. 2023). To facilitate sampling, it is common to center random variables when possible. In our implementation, all multivariate Gaussian distributions are implemented as a sum of the respective mean and a vector of independent, identical standard normal random variables, multiplied by the Cholesky factor of the respective covariance matrix.

PyMC has the advantage of a straightforward workflow to run the sampling algorithm on GPU hardware. We utilize the NumPyro implementation of NUTS (No-U-Turn Sampler; Phan, Pradhan, and Jankowiak 2019) and JAX (Just-in-time compilation for Accelerated numerical eXpressions; Bradbury et al. 2018), as described in the PyMC tutorials. A model run takes about one and a half hours on four Nvidia A30 GPUs. In each run, we start with 1000 warmup iterations and then generate 2000 samples in four chains with 500 draws each. For all model runs, the log-posterior plots indicate good mixing. The $\hat{R}$ statistic is below 1.1 for the large majority of random variables, which is consistent with the recommended convergence criterion (Vehtari et al. 2021). An exception are about 40 sample ages for the thermoremanent geomagnetic data, and in some cases, the associated predictions of direction or intensity. The largest observed $\hat{R}$ value for one of these ages is 2.05, followed by values below 1.6. Which and how many ages exactly are affected varies from model run to model run. This is likely due to ambiguity in the model, i.e. large dating uncertainties allow for multiple consistent combinations of age and field value, leading to multi modal posteriors. The posterior distributions for these ages (and predictions) should not be used for further analysis. Nilsson and Suttie (2021) describe a similar effect, and conclude that the overall results are not affected by this. For one run of the model that only contains magnetic data, we remove the affected observations. We rerun the model and by visual comparison found no impact on the outcome. The diagnostics also do not change. Following the method of Gelman et al. (2013), we find bulk and tail estimates of the effective sample size are larger than 1000 for the majority of quantities, but significantly lower than the optimal 2000 for most. To address this issue, which likely indicates mild autocorrelation, we keep only every second sample for our analysis.

The modeling code is available on github https://github.com/arthus701/radionuclides.

## Data

The input data to our model is put together from thermoremanent records of the geomagnetic field and radionuclide production rate data. The model is intended to cover the last 9000 years, until 7000 BCE. Geomagnetic records from this interval are queried from the GEOMAGIA database (Brown et al. 2015). The data selection process and uncertainty assignment are the same as in Nilsson and Suttie (2021). Radionuclide records are taken from two ice-core records for ^10^Be and from tree-ring measurements for ^14^C. The ice-core data stems from the Greenland Ice-Core Project (GRIP, Yiou et al. 1997; Muscheler et al. 2004; Vonmoos, Beer, and Muscheler 2006) and the European Project for Ice Coring in Antarctica, Dronning Maud Land (EDML, Steinhilber et al. 2012). Additional, shorter records are added to these two datasets, to bridge the gap between the long ice cores and the present. For Greenland we use short records from the North Greenland Ice Coring Project (NGRIP) record (Berggren et al. 2009), Dye-3 (Beer et al. 1990), and Milcent (Beer, Raisbeck, and Yiou 1991). For Antarctica, we use short records from Dome Fuji (Horiuchi et al. 2008), Siple Dome (Nishiizumi and Finkel 2007), and two different records from the South Pole (Raisbeck et al. 1990; Winski et al. 2019; Schaefer 2022). All records are averaged over consecutive 22-year bins, to obtain a common resolution for the only available long-term record from Antarctica (EDML). ^14^C atmospheric concentrations for the northern hemisphere are obtained from tree-ring measurements, compiled to construct the IntCal20 calibration curve (Reimer et al. 2020). These are also converted to production rates, similar to Nilsson et al. (2024). The ^14^C data are averaged over the same 22-year bins, to get a dataset consistent with the ^10^Be data. Except for studies of the 11-year cycle, where we use annual $\Delta ^{14}$C data (see below), the resulting production rate dataset is the same as used in Nilsson et al. (2024), and the same preprocessing is applied. All preprocessed datasets are provided together with the modeling code.

The combined dataset of radionuclide production rate and thermoremanent magnetic data covers multiple resolution regimes, that are represented by the different components in the model. The thermoremanent data can be used to resolve processes on centennial to millennial scale, while the 22-year averaged radionuclide records can be used to resolve faster processes. The resolution of the thermoremanent data is consistent with the average temporal dynamics of the large scale magnetic field (e.g. Christensen and Tilgner 2004). The 11-year Schwabe cycle in solar modulation cannot be resolved from the long term ice-core data. We consider a different dataset for our studies of this cycle, described in the following. Slower dynamics, like the occurrence of the Dalton and Maunder minima, which take place on timescales of 50 to 100 years, can be resolved from the radionuclide production rate data.

### Higher Resolution Data for 11-Year Cycle Recovery

To reconstruct annual global average ^14^C production rates over the last three millennia, we use tree-ring measurements of $\Delta ^{14}$C, defined as the per mil deviation of ^14^C/^12^C relative to a standard after correction for decay and fractionation (Stuiver and Polach 1977). To avoid artificial damping of the $\Delta ^{14}$C signal due to conflicting data from different laboratories we use exclusively annual $\Delta ^{14}$C data from Brehm et al. (2025) and Brehm et al. (2021) for the last millennium BCE (999 years) and the last millennium CE (968 years), respectively. To obtain a continuous $\Delta ^{14}$C dataset spanning the past 3500 years, for the remaining 1534 years, including gaps in the Brehm et al. (2025) and Brehm et al. (2021) datasets, we use $\Delta ^{14}$C data from the IntCal20 compilation (Reimer et al. 2020). The latter dataset contains 2097 partially overlapping measurements with temporal resolution ranging from annual measurements (55%) to 20-year averages (4%). Data after 1950 CE are heavily influenced by anthropogenic ^14^C produced during the nuclear bomb tests and are therefore not used. All data are combined to a continuous timeseries of $\Delta ^{14}$C using a Gaussian process with a prior mean function $m(t)$ and a Matérn- kernel. The IntCal20 $\Delta ^{14}$C dataset is constructed using Bayesian splines with a variable temporal resolution that adapts to the underlying data (Heaton et al. 2020). Based on comparisons to the power spectrum of the annual Brehm et al. (2021) data we find that the IntCal20 model is able to adequately capture $\Delta ^{14}$C variations on timescales of 40 years and longer, but appears overly damped on shorter timescales. We therefore define $m(t)$ as the IntCal20 model lowpass filtered with a cutoff frequency of 1/40 yrs^−1^. Based on inspection of the data residuals, after removing the prior mean, we assign a standard deviation of $\sigma =2$‰ and correlation time of $\tau =2$ years, for the Matérn- kernel function. The hyperparameters for the kernel function are chosen conservatively through model selection to preserve as much structure in the data as possible while avoiding excessive changes in the resulting ^14^C production rates. The $\Delta ^{14}$C variations are modeled at annual resolution using a Markov Chain Monte-Carlo (MCMC) approach. The likelihood function for the observations $o_{\Delta ^{14}C}$ is adapted according to the data resolution, i.e. a $\Delta ^{14}$C observation representing an average over the years $t_{1}$ to $t_{N}$ is modeled as

19 $$ o_{\Delta ^{14}C} = \frac{\sum _{i=1}^{N} \Delta ^{14}C(t_{i})}{N} + \epsilon _{\Delta ^{14}C} $$

where $\epsilon _{\Delta ^{14}C}\sim \mathcal{N}(0, \sigma _{\Delta ^{14}C})$ and $\sigma _{\Delta ^{14}C}$ is the measurement error. Individual samples from the MCMC model are then used to calculate ^14^C production rates similar to Nilsson et al. (2024), with 100 samples found to be enough to reach convergence.

## Results

**Figure 2:**
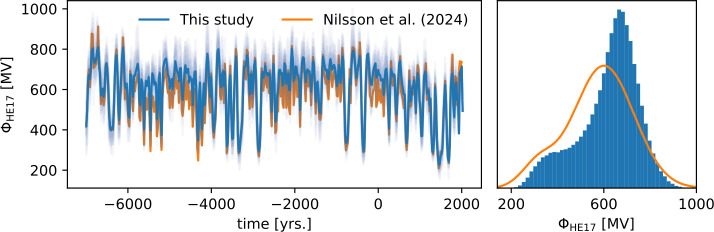
Reconstructed solar modulation from this study, using a model with no explicit long-term component. The left panel depicts the temporal evolution, with the mean drawn as a solid blue line and transparent blue lines showing samples from the posterior. An orange line shows the reconstruction of Nilsson et al. (2024). The right panel shows a histogram of all posterior samples at all knot points, except for the five most recent ones, where the model is “glued” to neutron monitor data. The Gaussian mixture fit of Nilsson et al. (2024) is added as an orange line, for comparison.

Figure 2 shows the reconstructed solar modulation based on the thermoremanent magnetic data and the 22-year average production rate data of ^14^C and ^10^Be. Note, that for comparison with other studies, we use a rescaled version of the solar modulation parameter. The production surfaces that enter our modeling are based on the local interstellar spectrum of Burger, Potgieter, and Heber (2000), parameterized by Usoskin et al. (2005). When presenting the results, we use the more recent and more accurate spectrum of Herbst, Muscheler, and Heber (2017). The conversion between the two is given by $\phi _{\text{HE17}} = 1.025 \cdot \phi + 24.18~\text{MV}$ (Herbst, Muscheler, and Heber 2017), where $\phi $ is the solar modulation based on Usoskin et al. (2005) (as used in the modeling).

The flexible prior in solar modulation leads to a clearly bimodal posterior. The lower mode, around 375 MV, reflects a low activity state and the upper mode, around 665 MV, reflects a state of regular activity. Based on the cumulative density corresponding to the right panel of Figure 2, the Sun spends about 10% of the time in a state of 380 MV or less. When fitting a mixture of two Gaussians to the distribution, we obtain parameters $\mu _{1}=375$ MV, $\sigma _{1}=80$ MV, $\mu _{2}=665$ MV, $\sigma _{2}=90$ MV and a relative weight of 18%. Nilsson et al. (2024) find a relative weight of 9%. Usoskin et al. (2014) report that during the last 3000 years, the Sun has spent 16% of its time in a low activity mode. The amount of bimodality of the model is controlled by a parameter $\kappa \in [0, 1]$, where higher values give higher bimodality. The prior mean of $\kappa $ is 0.125 with a standard deviation of 0.11. The posterior mean is 0.185, with a standard deviation of 0.05, i.e. the data pushes the model towards higher bimodality. In comparison to the reconstruction of Nilsson et al. (2024), shown as orange lines in Figure 2, the two modes of the histogram are more clearly separated. This is also reflected in the temporal evolution of the solar modulation parameter (Figure 2, right panel), where the level of the regular activity mode is slightly higher (by about 70 MV). The level of the low activity state agrees mostly with the reconstruction of Nilsson et al. (2024), except for the period between 6000 and 4000 BCE, where this study finds slightly higher levels (again, by about 70 MV). The comparatively low levels of $\phi $ in Nilsson et al. (2024) during this period might be an artefact of the Gaussian prior for $\phi $ that they used. During periods of moderately low activity (which are more shallow in Figure 2, between 450 and 600 MV), the Gaussian prior will push the regular activity mode of solar variability towards the prior mean (544 MV in Nilsson et al. (2024); see their Extended data Table 1). Different states of the Sun, reflecting in a multi-modal distribution of solar modulation, have been discussed before (e.g. Usoskin et al. 2014). Our reconstruction shows a more distinct separation of the low activity and the regular activity mode than other studies (e.g. Wu et al. 2018; Nilsson et al. 2024). While this can in part be attributed to the flexible prior distribution, it is also due to the better separation of geomagnetic field variations and solar modulation (compared to, e.g., Steinhilber et al. 2012; Usoskin et al. 2014). Snowball and Muscheler (2007) discuss how uncertainties in paleomagnetic intensity data limit the ability to reliably reconstruct solar variability over timescales beyond 1600 CE. The presented joint inversion in a Bayesian setting allows us to reflect data uncertainty in the model, but due to the limited resolution in paleomagnetic data, magnetic field variations on timescales faster than a hundred years may be missing in the model. Changes on this scale have however been observed only regionally (e.g. Gallet, Genevey, and Courtillot 2003; Shaar et al. 2022). Because the radionuclide production rates are modulated by the large scale magnetic field (dipole and axial quadrupole) only, the influence of these fast variations on the reconstructed variability is marginal.

### No Evidence for Long-Term Solar Variability

**Figure 3:**
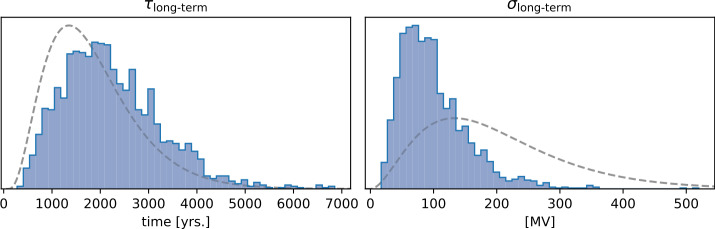
Histograms of the posterior distribution for the long-term correlation time (left) and the scale of the long-term zero mean Gaussian process (right). The prior distributions are added as dashed grey lines for comparison.

Pavón-Carrasco et al. (2018) suggest that variations in radionuclide production rates on the timescale of several hundred years are modulated by the Earth’s magnetic field. In line with this, Nilsson et al. (2024) report that such variations in the data have been misattributed to solar modulation (Hallstatt cycle) and can instead be explained by variability of the global geomagnetic field. To further investigate this, we include an explicit long-term component in our model. The long-term component is aimed to capture variations on the timescale of several hundred years and longer and is implemented as an additional zero mean Gaussian process. The scale (prior standard deviation) and correlation time are left as a hyperparameters. Figure 3 shows the prior and posterior distributions for the two hyperparameters of the long-term component. Evidently, the posterior correlation time is not well constrained by the data. We tried using a prior with more weight on shorter correlation times with no significantly different result. Additionally, we tried fixing the correlation time to 500, 1000 and 2000 years, also finding no different results. In all cases, the scale parameter has a posterior mean of about 60 MV, contracting considerably more than the correlation time. The fact that the posterior mean of the scale parameter is much lower than the prior mean (200 MV, see Table 2) indicates that the model tends to disregard the long-term component, i.e. the explicit long-term component is not necessary to explain the variability in the solar modulation.

**Figure 4:**
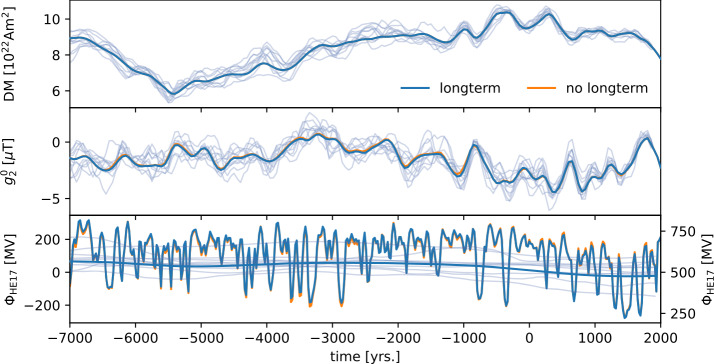
Geomagnetic dipole moment (top panel), axial quadrupole (center panel), and solar modulation (bottom panel) for a model with an explicit long-term component. Here we used 22-year averages for the production rate data. Samples from the posterior are drawn as transparent lines, to indicate the model spread. For comparison, the mean of the reconstruction with no explicit long-term component is shown in orange. In the bottom panel, the long-term component and the full (fast and long-term) reconstruction of the solar modulation are shown. The right scale is given for the full reconstruction. The left scale is adjusted to cover the same range, with the 0 MV line corresponding to the 500 MV line of the right scale. Posterior samples are drawn only for the long-term component. The solar modulation reconstruction of the model with no explicit long-term component (only fast variations) is drawn as an orange line.

Figure 4 shows the geomagnetic dipole moment (top panel), axial quadrupole (center panel), and solar modulation long-term component (bottom panel) retrieved from the extended model. Additionally, the reconstruction with no explicit long-term component is shown. The geomagnetic dipole moments are almost identical for the two. The 68%- and 95%-highest density intervals for the amplitudes of the longterm component are [0.00, 35.57] and [0.00, 72.72], respectively. When drawn on the same scale as the full reconstruction of the solar modulation, this basically gives straight lines (bottom panel of Figure 4). The individual samples show different constant means. These are highly correlated with the lower boundary parameter $\phi _{\text{lower}}$, which corresponds to a shift of the model that can also be realized by a constant mean in the long-term component. The small variations around the constant means seem to correspond to minor differences in the axial geomagnetic quadrupole. These differences are however well within the posterior uncertainties, indicating that the geomagnetic field reconstructions of the two models are indistinguishable. We conclude, that indeed the combination of thermoremanent records of the geomagnetic field and radionuclide production rate data provide no evidence for significant multi-centennial or longer scale variability in the solar modulation parameter. This is in contrast to earlier reconstructions that did not disentangle geomagnetic field and solar modulation variations and ignored hemispheric production differences that are relevant for the interpretation of polar ^10^Be records (Vonmoos, Beer, and Muscheler 2006; Steinhilber et al. 2012; Wu et al. 2018).

### Recovery of an 11-Year Cycle for the Last 3000 Years

Brehm et al. (2021) discuss the presence of an 11-year cycle in solar activity over the last millennium. To explore the possibility of recovering such a cycle within our modeling approach, we replace the last 3000 years of 22-year average ^14^C production rate data by annual data. We link this annual data to a quasi-periodic component in the prior. This component is added only to the likelihood term that models ^14^C-production rate for the last 3000 years, as for the earlier times and ^10^Be, 22-year average data is used, that cannot resolve the fast periodic signal.

**Figure 5:**
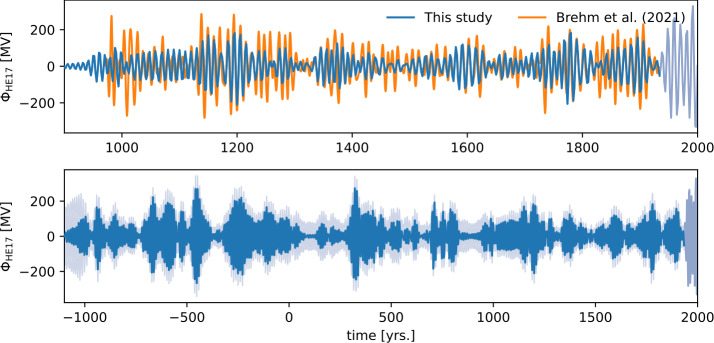
Six to 18-year band-pass filter of the reconstructed solar modulation with an explicit quasi-periodic component in the prior. Cascading second-order sections with a Butterworth filter design were used. The top panel shows the last 1000 years and the reconstruction of Brehm et al. (2021) for comparison (orange). For the period past 1934, the reconstruction is drawn in light blue, as it is inferred from reconstructions of the solar modulation parameter $\phi $ that are obtained from neutron monitor data (Nguyen et al. 2022, see also Section 2). The bottom panel shows our reconstruction for the last 3000 years (again band-pass filtered), together with the pointwise standard deviation as a shaded area.

Figure 5 shows the 6 to 18-year band-pass filtered signal of the posterior solar modulation with the explicit periodic component. Cascading second-order sections with a Butterworth filter design were used. The design and values were chosen to facilitate comparison with the reconstruction of Brehm et al. (2021), which is shown as well. Our reconstruction is in phase with that of Brehm et al. (2021), but shows a smaller amplitude, probably because of different carbon cycle models and different data treatment. Due to computational limitations, the calculated ensemble of ^14^C production rates are treated as independent annual observations (mean and standard deviation) in the model. This will be improved in future versions. For some times, the recovered cycle has a very low amplitude (year 800 to 950 CE) or almost disappears (around 100 CE). This is likely due to unavailability of annual data and large uncertainties in the underlying $\Delta ^{14}$C data, that result in a poor signal to noise ratio when converted to production rate data via a carbon cycle model. The signal to noise ratio can potentially be improved in a future work, by including high resolution ^10^Be. Still, the exploration shows the potential to recover an 11-year cycle within the presented approach.

### Thermoremanent Geomagnetic Data Do Not Constrain the Southern Hemisphere

One challenge when working with radionuclide production rate data is to provide an absolute scale for the data. To address this, our model includes free parameters that can slightly rescale the ^10^Be and ^14^C data. Recent studies of ^10^Be transport indicate, that the big majority of ^10^Be in the polar ice-core records is produced in the same hemisphere as the drill core (i.e. there is limited global mixing of ^10^Be, in contrast to e.g. ^14^C, which has a longer residence time; Zheng et al. 2024). Therefore, we include three individual scaling factors for the northern and southern hemisphere ^10^Be production rate records and the global ^14^C production rate record. A problem with this approach may be that the geomagnetic dataset has a strong bias towards the north, with very few records in the southern hemisphere, providing little constraint on the southern hemisphere production rates. To analyze the joint influence of the missing absolute scale and the bias in the geomagnetic dataset, we performed two additional model runs: one model that is based solely on the thermoremanent geomagnetic data and one model, where there is a common scaling for northern and southern hemisphere ^10^Be.

**Figure 6:**
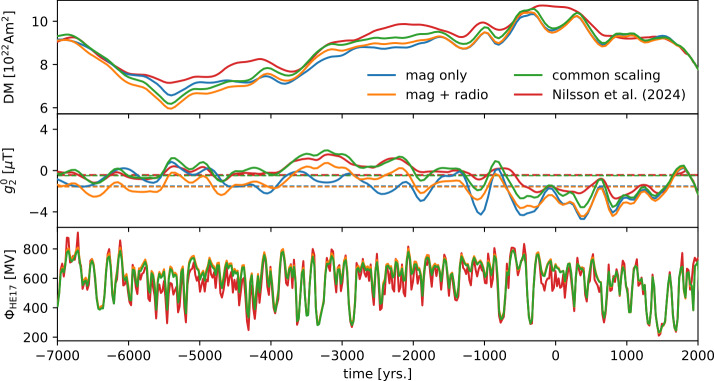
Geomagnetic dipole moment (top panel), axial quadrupole (center panel), and solar modulation (bottom panel) for different models. “mag only” refers to the model constructed solely from geomagnetic data, “mag + radio” to the model using both geomagnetic and radionuclide production rate data, and “common scaling” to the model using both datasets with a common scaling for the two ^10^Be datasets.

Even though the scaling factors are close to one for all models, the reconstructed geomagnetic field models show differences (Figure 6, top and center panel). Interestingly, the solar modulation is almost identical for the two models (Figure 6, bottom panel). The difference in the models appears to stem from balancing the dipole and quadrupole of the magnetic field. When leaving the scaling factors free for both ^10^Be datasets, the time average of the axial quadrupole is very close to the one for a model that is based solely on magnetic data (dashed lines in the center panel of Figure 6). It seems the scaling is used to reconcile the geomagnetic and production rate data. Using a common factor to scale the ^10^Be production rates leads to an axial quadrupole with an average close to zero and therefore a more symmetric field. The geomagnetic data are fit equally well by all three models. The resulting model is just as well compatible with the geomagnetic dataset, as the other models. Non-surprisingly, the geomagnetic dataset provides little constraint on the southern hemisphere and therefore on the southern hemisphere production rate data. The interplay of missing absolute scales for the production rate data and the poor coverage of the southern hemisphere by thermoremanent data limits the improvement of global geomagnetic field models by including production rate data. On the other hand, the fact that we see only a small improvement to the geomagnetic field reconstructions could be due to the fact that the data are largely consistent with each other. This is promising, as for geomagnetic field models on longer timescales, information from radionuclide production rates may be crucial.

## Conclusions

We present an approach to jointly invert solar modulation and a global magnetic field model from radionuclide production rate data and paleomagnetic data of thermoremanent origin. The prior for the solar modulation is constructed in a way that explicitly allows bimodality. Due to this flexibility, the reconstructed solar modulation shows clearly distinct modes of solar activity. A low activity mode, largely associated with the occurrence of grand solar minima, and a regular activity mode (Figure 2). In comparison to an earlier model by Nilsson et al. (2024), we find that the flexible prior as well as including the thermoremanent magnetic data directly further helps disentangling solar and geomagnetic signals. For epochs with moderately low activity, their model starts to resort to the Gaussian prior mean of 544 MV, which leads to an increase in dipole moment (see Figure 6, for example around 2000 BCE). This is possible, because in their model there is no geomagnetic field data from the southern hemisphere and dipole moment data are only included indirectly. The two modes of solar activity are more clearly separated than in earlier works (e.g. Steinhilber et al. 2012; Usoskin et al. 2014).

Extending the prior by an explicit long-term component aimed to model multi-centennial and longer variability in solar modulation yields no different results. The model disregards the long-term component (Figure 3), in contrast to other reconstructions of solar modulation, that show multi-centennial and longer variations (Vonmoos, Beer, and Muscheler 2006; Steinhilber et al. 2012; Wu et al. 2018). Instead, variations in solar modulation on this timescale can be explained by variations in the geomagnetic field, as was suggested earlier by Pavón-Carrasco et al. (2018). We explore the possibility to recover an 11-year cycle with the described modeling method by including high resolution ^14^C production rate data (Brehm et al. 2021, 2025) and adding a quasi-periodic component to the prior. The resulting model shows an 11-year cycle, but its presence is interrupted in intervals where no high resolution data are available. Finally, we examine how radionuclide data might improve magnetic field models, considering the influence of hemispherical bias and missing absolute scales in the production rate data. We find that due to the hemispherical bias, the improvements are limited.

## Data Availability

Original data is available from the cited studies and the GEOMAGIA database (Brown et al. 2015). The preprocessed data is available together with the modeling code via https://github.com/arthus701/radionuclides.
